# Representation of Patients With Chronic Kidney Disease in Clinical Trials of Cardiovascular Disease Medications: A Systematic Review

**DOI:** 10.1001/jamanetworkopen.2024.0427

**Published:** 2024-03-07

**Authors:** Julia M. T. Colombijn, Demy L. Idema, Sanne van Beem, Anna Marthe Blokland, Kim van der Braak, M. Louis Handoko, Linde F. Huis in ’t Veld, Tabea Kaul, Nurda Kolagasigil-Akdemir, Mike P. T. Kusters, Sabine C. A. Meijvis, Ilse J. Oosting, Rene Spijker, Michiel L. Bots, Lotty Hooft, Marianne C. Verhaar, Robin W. M. Vernooij

**Affiliations:** 1 Department of Nephrology and Hypertension, University Medical Center Utrecht, Utrecht, the Netherlands; 2 Julius Center for Health Sciences and Primary Care, University Medical Center Utrecht, Utrecht University, Utrecht, the Netherlands; 3 Cochrane Netherlands, Julius Center for Health Sciences and Primary Care, University Medical Center Utrecht, Utrecht University, Utrecht, the Netherlands; 4 Department of Cardiology, Amsterdam UMC, Vrije Universiteit Amsterdam, Amsterdam Cardiovascular Sciences, Amsterdam, the Netherlands; 5 Amsterdam Cardiovascular Sciences (Heart Failure and Arrhythmias), Amsterdam, the Netherlands; 6 Medical Library, Amsterdam UMC, University of Amsterdam, Amsterdam Public Health, Amsterdam, the Netherlands

## Abstract

**Importance:**

Patients with chronic kidney disease (CKD) are at high risk for cardiovascular disease, but their systematic underrepresentation in cardiovascular randomized clinical trials (RCTs) limits the generation of appropriate evidence to guide cardiovascular risk management (CVRM).

**Objective:**

To evaluate the underrepresentation of patients with CKD in cardiovascular RCTs, and to highlight evidence gaps in CVRM medications in this population.

**Evidence Review:**

A systematic search was conducted in ClinicalTrials.gov from February 2000 through October 2021 for RCTs with full-text publications. If no full-text publications were found in ClinicalTrials.gov, MEDLINE, Embase, and Google Scholar were also searched. Eligible RCTs were those evaluating the effectiveness of antiplatelets, anticoagulants, blood pressure–lowering drugs, glucose-lowering drugs, or cholesterol-lowering drugs in adults with cardiovascular disease or cardiovascular risk factors. Trials with a sample size of fewer than 100 patients were excluded.

**Findings:**

In total, 1194 RCTs involving 2 207 677 participants (mean [SD] age, 63 [6] years; 1 343 970 males [64%]) were included. Since 2000, the percentage of cardiovascular RCTs excluding patients with CKD has increased from 66% to 79% (74% overall [884 RCTs]). In 864 RCTs (72%), more patients were excluded than anticipated on safety grounds (63% [306] of trials required no dose adjustment, and 79% [561] required dose adjustment). In total, 158 RCTs (13%) reported results for patients with CKD separately (eg, in subgroup analyses). Significant evidence gaps exist in most CVRM interventions for patients with CKD, particularly for those with CKD stages 4 to 5. Twenty-three RCTs (2%) reported results for patients with an estimated glomerular filtration rate less than 30 mL/min/1.73 m^2^, 15 RCTs (1%) reported for patients receiving dialysis, and 1 RCT (0.1%) reported for recipients of kidney transplant.

**Conclusions and Relevance:**

Results of this systematic review suggest that representation of patients with CKD in cardiovascular RCTs has not improved in the past 2 decades and that these RCTs excluded more patients with CKD than expected on safety grounds. Lack of reporting or underreporting of results for this patient population is associated with evidence gaps in the effectiveness of most CVRM medications in patients with all stages of CKD, particularly CKD stages 4 to 5.

## Key Points

**Question:**

How often are patients with chronic kidney disease (CKD) excluded in cardiovascular randomized clinical trials (RCTs), and what are the evidence gaps in cardiovascular medications for these patients?

**Findings:**

In this systematic review of 1194 RCTs involving over 2 million patients, the proportion of RCTs that excluded patients with CKD increased in the past 20 years. Such RCTs typically excluded more patients than expected on safety grounds.

**Meaning:**

Findings of this study suggest that lack of RCTs reporting results for patients with CKD plays a role in the significant evidence gaps in the effectiveness of cardiovascular disease medications for patients with all stages of CKD, especially stages 4 to 5.

## Teaser

This systematic review describes representation of adults with chronic kidney disease (CKD) in randomized clinical trials for antithrombotic, antihypertension, glucose-lowering, and cholesterol-lowering drugs from 2000 to 2021.

## Introduction

Chronic kidney disease (CKD) affects almost 700 million people worldwide and is the cause of 1.9 million cardiovascular deaths annually.^1,2^ Over 60% of patients with CKD have a history of cardiovascular disease (CVD), which is also the main cause of death in this population.^3,4^ Almost all patients with CKD have a much higher risk for CVD than kidney failure.^5,6^ This elevated CVD risk is already observed for patients with an estimated glomerular filtration rate (eGFR) less than 75 mL/min/1.73 m^2^ and increases as CKD progresses independent of other risk factors, such as hypertension and diabetes.^3,7^

The high cardiovascular risk in patients with CKD underscores the importance of effective cardiovascular risk management (CVRM) for these patients. Nevertheless, even though over 90% of patients with CKD are prescribed CVRM medications, evidence is limited on the safety and effectiveness of these medications in this population.^8,9^ Historically, patients with CKD largely have been underrepresented in cardiovascular randomized clinical trials (RCTs). They are frequently excluded due to concerns about the safety and effectiveness of interventions. Even the RCTs without explicit CKD exclusion criteria often do not include these patients nor assess treatment effects for them.^10,11,12,13^

Lack of information about the effectiveness of CVRM medications in patients with CKD undermines effective CVRM. Effectiveness estimates about CVRM medications from RCTs that excluded patients with CKD cannot be extrapolated carelessly since the increased CVD risk in patients with CKD and altered pathophysiological processes of CVD can modify the effectiveness of treatments.^14^ As CKD progresses to kidney failure, patients’ CVD burden shifts from atherosclerotic CVD to medial arterial calcification, cardiac arrhythmias, left-ventricular hypertrophy, and sudden cardiac death.^14^ A higher cardiovascular risk could enhance the effectiveness of CVRM for patients with CKD because a greater absolute risk reduction can be achieved. However, lower life expectancy and the induction of additional pathways in CVD pathophysiological processes, which are not inhibited by traditional CVRM medications, could offset these benefits and render treatment futile.^15,16^

Several systematic reviews, which included RCTs published up to 2014, have reported on the underrepresentation of patients with CKD in cardiovascular RCTs.^10,11,12,13^ However, it is unclear whether the representation of patients with CKD in cardiovascular RCTs has improved over the past years and whether this population has been included in RCTs evaluating the effectiveness of new treatments, such as sodium-glucose cotransporter 2 (SGLT2) inhibitors and direct oral anticoagulants (DOACs). Furthermore, the systematic exclusion of patients with CKD makes it difficult to ascertain which CVRM medications have available evidence on their effectiveness and safety, specifically for people with CKD. An overview of the RCTs evaluating the effectiveness of CVRM medications for patients with different stages of CKD is currently lacking. Therefore, this systematic review aimed to evaluate the underrepresentation of patients with CKD in cardiovascular RCTs in the past 20 years and to highlight evidence gaps in CVRM medications in this population.

## Methods

This systematic review is registered prospectively in the PROSPERO International Prospective Register of Systematic Reviews (CRD42022296746). The full protocol has been published previously.^17^ We followed the Preferred Reporting Items for Systematic Reviews and Meta-analyses (PRISMA) reporting guideline.

### Data Sources and Searches

ClinicalTrials.gov was searched through the Cochrane Central Register of Controlled Trials from inception (February 2000) through October 2021 using a combination of keywords for CVD, cardiovascular risk factors, and included interventions to identify planned, ongoing, terminated, and completed RCTs. Full-text publications were retrieved up to May 2023 from ClinicalTrials.gov. If no full-text publications were found in ClinicalTrials.gov, MEDLINE, Embase, and Google Scholar were also searched to retrieve full texts. Trial records were excluded if no publications could be found. Landmark RCTs that were not identified in the search were added manually.

### Study Selection

Two reviewers (including J.M.T.C., D.L.I., S.V.B., A.M.B., K.V.D.B., N.K.A., I.J.O., R.W.M.V.) screened clinical trial records and publications independently based on the eligibility criteria. Disagreements were resolved by discussion.

Eligible RCTs were those that evaluated the association of antiplatelets, anticoagulants, blood pressure–lowering drugs, glucose-lowering drugs, or cholesterol-lowering drugs, which are recommended by the European Society of Cardiology, the American Heart Association, the American Stroke Association, the American College of Cardiology, and the American Diabetes Association for the prevention of CVD,^18,19,20,21,22,23,24,25,26,27,28,29,30,31,32,33,34,35,36,37,38,39^ with all-cause or cardiovascular mortality, CVD (as composite end points and individual events), peripheral arterial disease, or kidney failure in adults with a history of CVD or 1 or more CVD risk factors. In these RCTs, interventions were compared with placebo, usual care, another therapy, or a different treatment dose or duration. Trials with a sample size of fewer than 100 patients were excluded.

### Data Extraction and Quality Assessment

Data extraction was performed by 1 reviewer (including D.L.I., S.V.B., A.M.B., K.V.D.B., L.F.H.I.V., T.K., N.K.A., M.P.T.K., I.J.O.) using a standardized form and was verified by another reviewer (J.M.T.C.). A list of extracted variables is described in the protocol.^17^ Risk of bias was not assessed since bias in study design is unlikely to affect whether patients with CKD are excluded from RCTs or whether authors report results for these patients (as a subgroup analysis or by restriction of the study population).

### Statistical Analysis

Outcomes of interest were the frequency of excluding patients with CKD and reporting results for patients with CKD through subgroup analyses or restriction of the study population. Exclusion of patients with CKD was defined as the exclusion of patients meeting kidney-related eligibility criteria. If RCTs did not specify kidney-related eligibility criteria, we presumed these patients were not excluded.

Categorical variables were described as frequency (percentage), and continuous variables were described as mean (SD) if they followed a normal distribution or as median (IQR) otherwise. The frequency of excluding patients with CKD was evaluated for different periods, medications, and dose recommendations for patients with CKD. Dose recommendations were categorized based on *The Renal Drug Handbook* as follows: no dose adjustment, dose adjustment in CKD stage 3 or stages 4 to 5, and contraindication in CKD stages 4 or 5.^40^ An overview of RCTs published for patients with different stages of CKD was visualized in an evidence map. Data analysis was performed with R, version 4.3 (R Project for Statistical Computing).

## Results

Overall, 1194 RCTs involving 2 207 677 participants were included (eFigure 1 in Supplement 1). The search identified 13 017 RCTs, of which 8780 were excluded. Of 1419 RCTs, no full text could be retrieved. The remaining 2818 records were screened on full text. The main reasons for exclusion were no outcomes of interest (n = 884), wrong intervention (n = 304), and insufficient sample size (n = 77). Included RCTs (n = 1194) had a median (IQR) follow-up of 24.0 (12.0-39.6) months, and 81 trials (7%) had published a protocol only. Glucose-lowering drugs were evaluated in 552 RCTs (46%), antiplatelets and anticoagulants in 229 RCTs (19%), blood pressure–lowering drugs in 221 RCTs (19%), and a combination of these interventions in 30 RCTs (3%) (Table).

**Table.** Characteristics of Included Randomized Clinical Trials (RCTs)

Variables	RCTs, No. (%)		
	Total (n = 1194)	Excluding patients with CKD (n = 884)	Not excluding patients with CKD (n = 310)
**Study characteristics**			
Year of publication			
Before 2000	21 (2)	12 (1)	9 (3)
2000-2005	50 (4)	33 (4)	17 (5)
2006-2010	239 (20)	173 (20)	66 (21)
2011-2015	393 (33)	286 (32)	107 (34)
2016-2020	418 (35)	322 (36)	96 (31)
After 2020	73 (6)	58 (7)	15 (5)
Sample size			
Total	2 207 677	1 595 831	563 038
Median (IQR)	568 (304-1315)	559 (311-1250)	530 (280-1288)
Follow-up, median (IQR), mo	24.0 (12.0-39.6)	26.4 (12.0-40.8)	17.8 (5.5-30.0)
Published only a protocol	81 (7)	64 (8)	17 (5)
Location			
Multicontinental	576 (48)	443 (50)	133 (43)
Europe	165 (14)	117 (13)	48 (16)
North America	172 (14)	116 (13)	53 (17)
Asia, Australia, or Africa	281 (24)	208 (24)	73 (24)
Funding source^a^			
Industry	987 (83)	736 (83)	251 (81)
Government	81 (7)	57 (8)	24 (6)
Institution	151 (13)	110 (12)	41 (13)
Unspecified or miscellaneous	49 (4)	35 (4)	14 (5)
Type of intervention			
Blood pressure–lowering drugs	221 (19)	182 (20)	39 (13)
Glucose-lowering drugs	552 (46)	424 (48)	128 (41)
Cholesterol-lowering drugs	162 (14)	126 (15)	36 (12)
Antiplatelets or anticoagulants	229 (19)	132 (15)	97 (31)
Combination of interventions	30 (3)	20 (2)	10 (3)
Dose adjustment or contraindication for CKD	706 (59)	578 (65)	128 (41)
**Participant characteristics**			
Age, mean (SD), y	63 (6)	64 (6)	62 (6)
Sex			
Female	747 390 (36)	571 344 (27)	176 046 (8)
Male	1 343 970 (64)	990 419 (47)	353 551 (17)
eGFR, mL/min/1.73 m^2^^b^			
Mean (SD)	73 (13)	73 (13)	77 (10)
Not reported	899 (75)	623 (71)	276 (89)
≥90	79 (27)	74 (28)	5 (14)
60-89	169 (57)	144 (55)	25 (73)
45-59	27 (9)	24 (9)	3 (8)
30-44	13 (4)	13 (5)	0
15-29	7 (2)	6 (3)	1 (3)
Serum creatinine, mg/dL^b^			
Median (IQR)	1.00 (0.96-1.04)	1.00 (0.97-1.04)	1.00 (0.95-1.01)
Not reported	1040 (87)	750 (85)	290 (94)
<1.0	77 (50)	67 (50)	10 (50)
1.0-1.49	61 (40)	51 (38)	10 (50)
1.5-2.00	9 (6)	9 (7)	0
>2.0	7 (5)	7 (5)	0
Dialysis	17 (<1)	17 (2)	0
Transplant	1 (<1)	1 (<1)	0

Abbreviations: CKD, chronic kidney disease; eGFR, estimated glomerular filtration rate.

SI conversion factor: To convert eGFR to milliliter per second per square meter, multiply by 0.0167; serum creatinine to micromoles per liter, multiply by 88.4.

^a^ Because RCTs can have multiple funding sources, percentages do not add up to 100 percent.

^b^ Percentages only provided for proportion of patients without missing eGFR or serum creatinine data.

Participants had a mean (SD) age of 63 (6) years and included 747 390 females (36%) and 1 343 970 males (64%); 80 trials had missing data on sex (n = 116 317). The mean (SD) eGFR was 73 (13) mL/min/1.73 m^2^ and the median (IQR) serum creatinine level was 1.00 (0.96-1.04) mg/dL, but these variables were reported in only 295 (25%) and 154 (13%) RCTs, respectively. Patients receiving dialysis were included in 17 RCTs (<1%), and recipients of a kidney transplant were included in 1 RCT (<1%). An overview of included RCTs and their characteristics are provided in eTables 1 to 10 in Supplement 2.

### Underrepresentation of Patients With CKD

**Figure 1.**
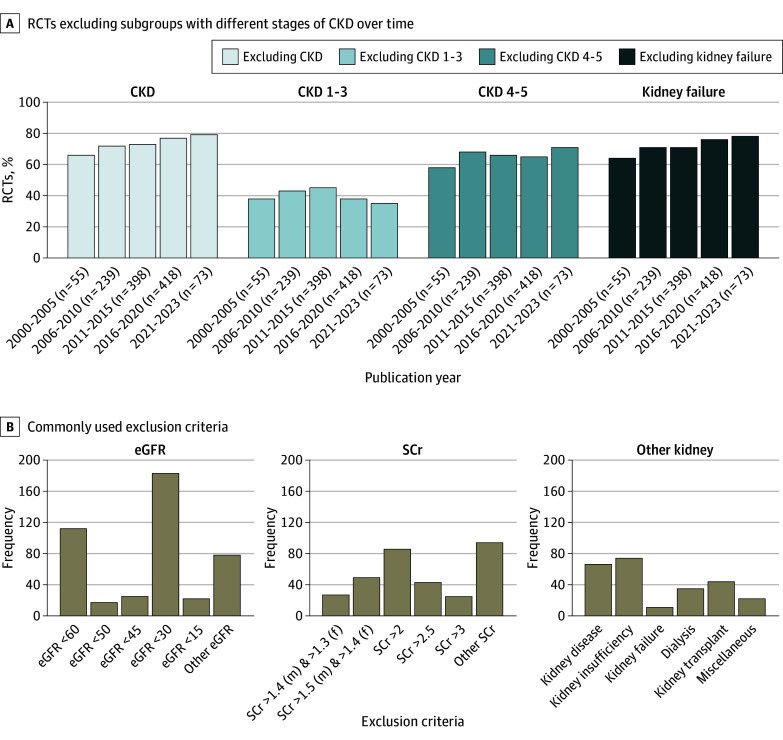
Overview of Exclusion of Patients With Chronic Kidney Disease (CKD) From Cardiovascular Randomized Clinical Trials (RCTs) eGFR indicates estimated glomerular filtration rate; SCr, serum creatinine.

**Figure 2.**
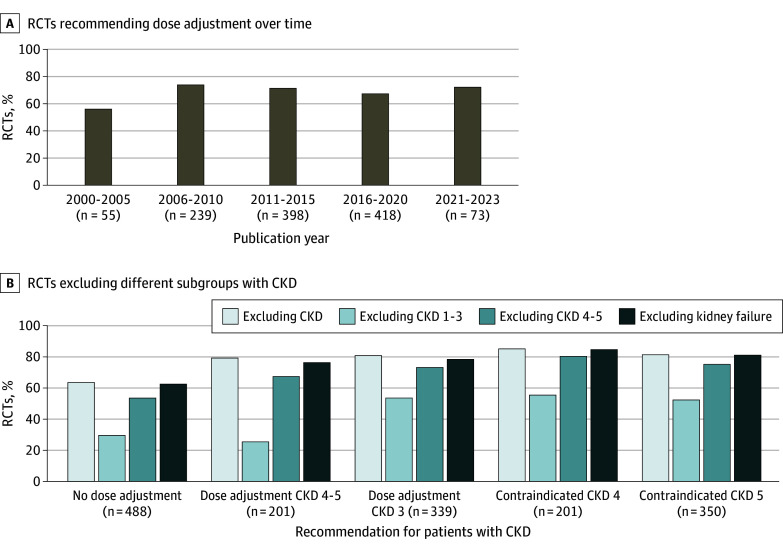
Exclusion of Patients With Chronic Kidney Disease (CKD) Stratified by Prescription Recommendations RCT indicates randomized clinical trial.

Since 2000, the percentage of RCTs excluding subgroups of patients with CKD has increased from 66% to 79% (74% overall [884 RCTs]) (Figure 1A). Patients with an eGFR greater than 30 mL/min/1.73 m^2^, serum creatinine level less than 2 mg/dL, or a history of CKD (hereafter, CKD stages 1-3) were excluded from 458 RCTs (38% of all included RCTs, and 52% of RCTs that excluded patients with CKD) (Figure 1A). In the past 20 years, patients with CKD stages 4 to 5 have been excluded from cardiovascular RCTs more frequently, whereas the exclusion of patients with CKD stages 1 to 3 has remained stable (Figure 1A). The proportion of RCTs in which dose adjustment based on kidney function was required or medication was contraindicated based on kidney function remained consistent across different periods (eg, 2000-2005 to 2021-2023: 38% to 35% for CKD stages 1-3; 58% to 71% for CKD stages 4-5) (Figure 2A; eFigure 3 in Supplement 1). The kidney exclusion criteria applied were heterogeneous but generally based on eGFR (442 RCTs [50%]) or serum creatinine level (324 RCTs [37%]) (Figure 1B). The exclusion of patients with CKD for individual drug groups is illustrated in eFigure 2 in Supplement 1.

In 864 RCTs (72%), more patients with CKD were excluded than expected on safety grounds. Patients with CKD were excluded in 306 of 488 RCTs (63%) in which no dose adjustment for the interventions on kidney function was required. The rate of exclusion of patients with CKD was over 80% in RCTs in which dose adjustments based on kidney function were necessary or interventions were contraindicated based on kidney function. However, 561 of 706 RCTs (79%) also excluded more patients with CKD than necessary on safety grounds (Figure 2B; eFigure 3 in Supplement 1).

### Evidence and Evidence Gaps in CVRM Medications in Patients With CKD

**Figure 3.**
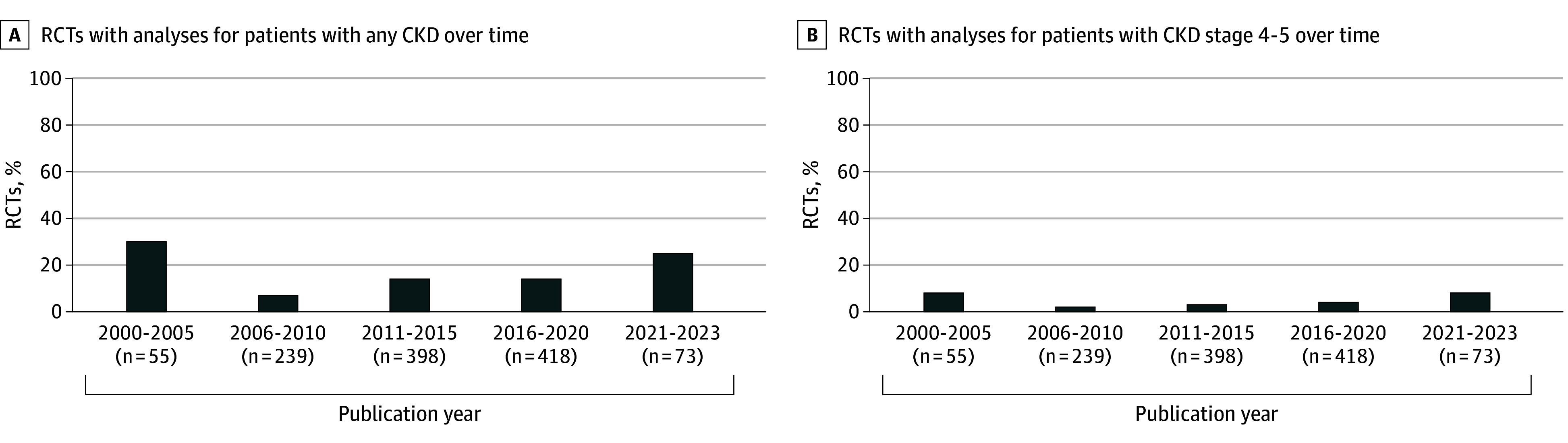
Percentage of Randomized Clinical Trials (RCTs) With Analyses for Patients With Any or Stages 4 to 5 Chronic Kidney Disease (CKD)

**Figure 4.**
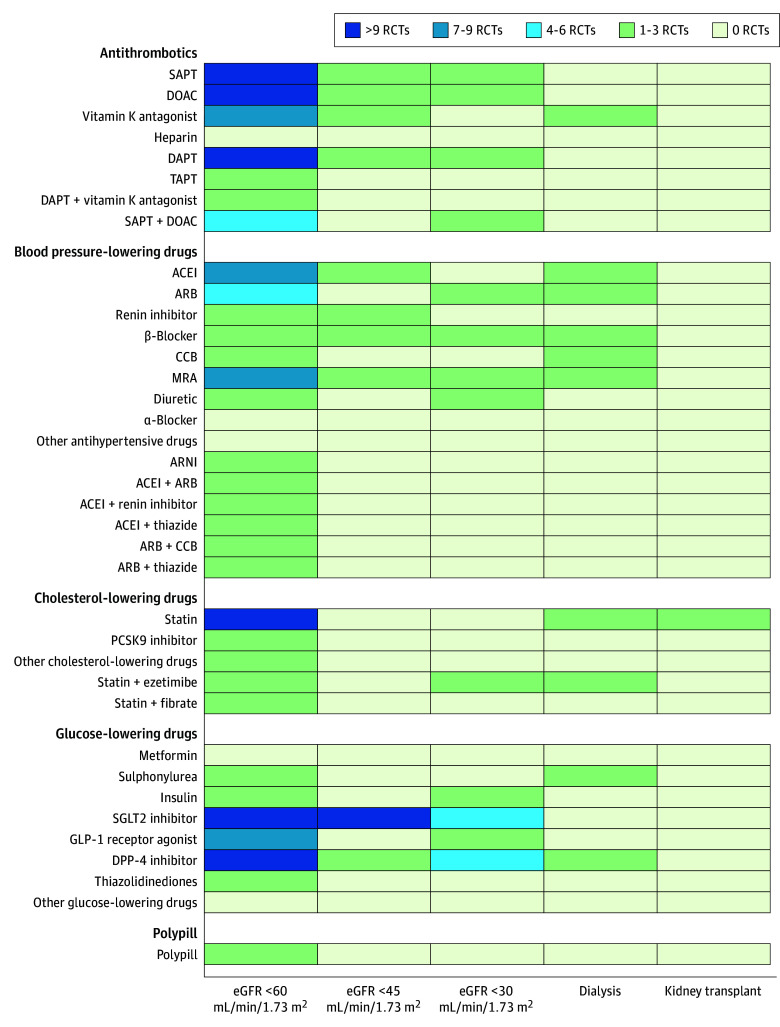
Heat Map of Analyses for Major Adverse Cardiovascular Events for Patients With Different Stages of Chronic Kidney Disease (CKD) ACEI indicates angiotensin-converting enzyme inhibitor; ARB, angiotensin receptor blocker; ARNI, angiotensin receptor–neprilysin inhibitor; CCB, calcium channel blocker; DAPT, double antiplatelet therapy; DOAC, direct oral anticoagulant; DPP-4 inhibitor, dipeptidyl peptidase 4; eGFR, estimated glomerular filtration rate; GLP-1 receptor agonist, glucagon-like peptide 1; MRA, mineralocorticoid receptor antagonist; PCSK9 inhibitor, proprotein convertase subtilisin/kexin type 9; RCT, randomized clinical trial; SAPT, single antiplatelet therapy; SGLT2 inhibitor, sodium-glucose cotransporter 2; TAPT, triple antiplatelet therapy.

In total, 158 RCTs (13%) reported results for patients with CKD. Of these RCTs, 34 (3%) included patients with CKD only (4 cholesterol-lowering drugs, 13 blood pressure–lowering drugs, 15 glucose-lowering drugs, and 2 antithrombotic drugs). Twenty-three RCTs (2%) reported results for patients with an eGFR less than 30 mL/min/1.73 m^2^, 15 RCTs (1%) reported for patients receiving dialysis, and 1 RCT (0.1%) reported for recipients of kidney transplant. The percentage of RCTs that reported results for patients with CKD has not increased in the past 20 years, from 30% in 2000 to 2005 to 25% in 2021 to 2023 (Figure 3). Analyses for patients with CKD were predominantly performed for composite cardiovascular end points (112 RCTs [66%]) in heterogeneous strata (Figure 4; eFigures 4 and 5 in Supplement 1). Few RCTs conducted analyses for individual cardiovascular end points, particularly for heart failure, peripheral arterial disease, and kidney failure (eFigures 4 and 6-12 in Supplement 1). The mean (SD) eGFR in RCTs that conducted subgroup analyses was 71 (12) mL/min/1.73 m^2^, but this parameter was reported in 102 of 171 RCTs (60%).

We identified significant evidence gaps in CVRM medications for all patients with CKD. Evidence gaps were most notable for patients with CKD stages 4 to 5. An overview of analyses for end points other than major adverse cardiovascular events (MACE) is provided in eFigures 6 to 11 in Supplement 1.

### Blood Pressure–Lowering Drugs

Most RCTs (26 of 52 [50%]) that evaluated blood pressure–lowering drugs preventing MACE for patients with CKD focused on angiotensin-converting enzyme inhibitors, angiotensin receptor blockers, and mineralocorticoid receptor antagonists (Figure 4; eFigure 5 in Supplement 1). Each antihypertensive drug was evaluated for patients with an eGFR less than 60 mL/min/1.73 m^2^, except for α-blockers. Angiotensin receptor blockers, β-blockers, and mineralocorticoid receptor antagonists were assessed for patients with an eGFR less than 30 mL/min/1.73 m^2^ and patients receiving dialysis. Angiotensin-converting enzyme inhibitors and calcium channel blockers were also evaluated for patients receiving dialysis and thiazides for patients with an eGFR less than 30 mL/min/1.73 m^2^. Other antihypertensives were not evaluated in these populations. None of the antihypertensives were evaluated for recipients of a kidney transplant (Figure 4; eFigure 5 in Supplement 1).

### Cholesterol-Lowering Drugs

The effectiveness of cholesterol-lowering drugs for preventing MACE in patients with CKD was evaluated almost exclusively for statins (Figure 4; eFigure 5 in Supplement 1). Statins were evaluated for patients with an eGFR less than 60 mL/min/1.73 m^2^ as monotherapy or in combination with ezetimibe. Proprotein convertase subtilisin/kexin type 9 inhibitors, niacin, and icosapent ethyl were also evaluated for this population. For patients with an eGFR less than 30 mL/min/1.73 m^2^, only the combination of statins and ezetimibe was evaluated. For patients receiving dialysis, statins were evaluated as monotherapy and in combination with ezetimibe. For kidney transplant recipients, only statin monotherapy was evaluated (Figure 4; eFigure 5 in Supplement 1).

### Antiplatelets and Anticoagulants

Of all CVRM medications, antiplatelets and anticoagulants were studied most frequently for patients with CKD (Figure 4; eFigure 5 in Supplement 1). The effectiveness of single antiplatelet therapy (SAPT; 17 of 55 RCTs [31%]), double antiplatelet therapy (DAPT; 13 [24%]), and DOACs (12 [22%]) was evaluated for patients with CKD in multiple RCTs. However, few of these RCTs reported results for patients with an eGFR less than 45 mL/min/1.73 m^2^ (Figure 4; eFigure 5 in Supplement 1). For patients with an eGFR less than 30 mL/min/1.73 m^2^, the effectiveness of SAPT, DAPT, DOACs, and DOACs plus SAPT was evaluated. For patients receiving dialysis, evidence was limited to the comparison of DOACs with vitamin K antagonists, and the effectiveness of antiplatelets was not evaluated at all. None of the RCTs evaluated the effectiveness of antiplatelets and anticoagulants for recipients of a kidney transplant (Figure 4; eFigure 5 in Supplement 1).

### Glucose-Lowering Drugs

The effectiveness of SGLT2 inhibitors (13 of 51 RCTs [25%]), glucagon-like peptide 1 (GLP-1) receptor agonists (10 [20%]), and dipeptidyl peptidase 4 (DPP-4) inhibitors (6 [12%]) for preventing MACE for patients with CKD was evaluated in multiple RCTs. However, hardly any evidence was available for older glucose-lowering drugs, such as metformin, sulphonylureas, and insulin (Figure 4; eFigure 5 in Supplement 1). Similar to antiplatelets and anticoagulants, there were little data for patients with an eGFR less than 30 mL/min/1.73 m^2^. For these patients, the effectiveness of SGLT2 inhibitors, DPP-4 inhibitors, GLP-1 receptor agonists, and insulin was evaluated. For patients receiving dialysis, DPP-4 inhibitors and sulphonylureas were compared. None of the glucose-lowering drugs were assessed in recipients of a kidney transplant (Figure 4; eFigure 5 in Supplement 1).

## Discussion

In this systematic review, we found no improvement in the representation in RCTs of patients with CKD over the past 2 decades. On the contrary, since 2000, the number of cardiovascular RCTs that excluded subgroups of patients with CKD has increased. Exclusion criteria were heterogeneous and cardiovascular RCTs consistently excluded a larger number of patients with CKD than would be anticipated on safety grounds. In addition, only 13% of included cardiovascular RCTs evaluated the effectiveness of CVRM medications for patients with CKD, mostly in subgroup analyses. Although for almost all medications some data were published for patients with CKD stage 3, there were evidence gaps across all CVRM medications and patients with all stages of CKD, particularly stages 4 to 5.

The persistently high proportion of RCTs that excluded patients with CKD in the past 20 years cannot be attributed solely to safety concerns. Although the absolute number of RCTs requiring dose adjustment on kidney function for CVRM medications increased in this period, the proportion of RCTs requiring such adjustment remained stable. While excluding patients with CKD from RCTs due to safety concerns can be justifiable, the substantially more stringent kidney exclusion criteria compared with prescription thresholds in clinical practice suggest there were additional reasons for excluding patients with CKD. Practical issues, such as the necessity for dose adjustments, concerns about heterogeneity in treatment effects, or limited life expectancy, could also discourage investigators from including patients with CKD in their RCTs.

The evidence gaps for patients with CKD in cardiovascular RCTs can be traced back to the ongoing widespread exclusion of this population. Between 1980 and 2005, 56% to 76% of cardiovascular RCTs excluded patients with CKD.^10,11^ The representation of patients with CKD has not improved after this period with reported rates of exclusion, including this study, ranging from 46% to 79%.^12,13^ These rates likely underestimate the underrepresentation of patients with CKD because RCTs without explicit exclusion criteria may not enroll an adequate proportion of participants with CKD. Excluding patients with CKD due to possible treatment heterogeneity or initial safety concerns does not necessarily lead to evidence gaps, provided that separate RCTs are conducted to assess the effectiveness of medications for patients with CKD. However, in practice, just 3% of included cardiovascular RCTs were conducted specifically for patients with CKD. Although the proportion of RCTs that reported results for patients with CKD has increased from 8% of the RCTs published between 1980 and 2005,^11^ only 25% of RCTs published after 2020 reported results of patients with CKD.

### Implications for Practice 

Currently, most cardiovascular RCTs that reported results for patients with CKD focused on those with CKD stage 3. For patients with CKD stages 4 to 5, which compose 10% of patients with CKD (ie, 85 million patients),^41^ analyses were often absent, particularly for recipients of a kidney transplant. The lack of RCTs assessing the effectiveness of CVRM medications for patients with CKD means that, in practice, practitioners must resort to extrapolating results from RCTs conducted in other populations, assuming that the treatment effects are comparable. However, this assumption is increasingly less likely to hold for patients with more advanced CKD stages where CKD-specific risk factors like vascular calcification, uremia, chronic inflammation, and immunosuppressive therapy to prevent graft rejection, combined with high risk and reduced life expectancy, can modify the treatment effect.^42,43^

The complexity of extrapolating results to patients with CKD was illustrated by statins. Although these drugs reduced cardiovascular risk in patients with an eGFR less than 60 mL/min/1.73 m^2^, their effectiveness has not been demonstrated in individuals with kidney failure.^44^ The lack of RCTs conducted in patients between these ends of the CKD spectrum makes it impossible to determine the tipping point at which statins lose their benefits. Consequently, patients may unintentionally be overtreated or undertreated since the balance between benefits and adverse effects remains unknown.

In addition to an absolute lack of RCTs, limitations in the analyses further hamper CVRM treatment for patients with CKD. Heterogeneity in exclusion criteria and inadequate reporting of baseline kidney function are associated with reduced comparability of RCTs, whereas the small sample size of strata plays a role in underpowered analyses and imprecision in effect size estimates. Furthermore, the lack of RCTs evaluating the association of CVRM medications with individual cardiovascular end points and kidney end points in patients with CKD means that the effectiveness of these drugs for these individual end points remains unknown. Stratifying RCT cohorts breaks randomization and can introduce confounding due to clustering of other CVD risk factors in patients with CKD.^45,46^ These limitations are likely to amount in a GRADE (Grading of Recommendations, Assessment, Development, and Evaluations) of low or very low certainty of evidence for most CVRM medications in patients with CKD, meaning that their effectiveness might be markedly different from the estimated treatment effect.^47^

### Implications for Research

The increasing prevalence of CKD (including dialysis and transplant), widespread prescription of CVRM medications, and uncertainty about the effectiveness of various CVRM medications in patients with CKD underscore the urgency of adequate representation of this population in cardiovascular RCTs.^48,49^ Despite efforts of the US Food and Drug Administration and European Medicines Agency to promote the enrollment of patients with CKD and numerous reviews and editorials addressing this issue, the representation of patients with CKD in cardiovascular RCTs has not increased in the past 40 years.^12,13,50,51^ Moreover, although we found a significant underrepresentation of patients with CKD in cardiovascular RCTs, the results likely underestimated the actual underrepresentation of patients with CKD because RCTs without explicit exclusion criteria may not enroll an adequate proportion of patients with CKD, only aggravating the problem.

Bridging the evidence gap for treatment of cardiovascular risk in patients with CKD requires the collaboration among different stakeholders, including pharmaceutical companies, medication regulatory authorities, scientific societies, funding bodies, and clinical steering committees, and starts with the adequate documentation of kidney function and disease as well as proportional inclusion of patients with CKD to enable separate analyses for patients with vs without kidney disease or patients with different stages of kidney disease. New evidence for patients with CKD stages 4 to 5 (including those receiving kidney replacement therapy) should be prioritized, considering that the evidence gaps are largest for this population. Despite the challenges of including patients with CKD stages 4 to 5 or conducting separate RCTs for them, analyses to obtain reliable estimates on the effectiveness of cardiovascular medications in patients with CKD are only feasible if a sufficient number of these patients are included in RCTs. Additionally, more evidence is needed on the effectiveness of CVRM medications for individual cardiovascular and kidney end points. Innovative RCT designs, such as adaptive platform trials based on a master protocol, might be a means to rapidly generate evidence for a range of treatment strategies for different groups of patients with CKD. Furthermore, emulated target trials with clinical data present another opportunity to fill evidence gaps in the effectiveness of CVRM medications in patients with CKD, especially for drugs regularly prescribed in practice where conducting new RCTs is prohibitively expensive and time consuming.^52^

### Limitations

This study has several limitations. We might have underestimated the exclusion of patients with CKD in RCTs with ambiguous exclusion criteria, such as chronic disease or life-limiting disease, and might have missed RCTs that were not registered in ClinicalTrials.gov. However, we are confident that the number of missed RCTs was small since the sensitivity of searches in trial registries and electronic databases has been demonstrated to be comparable.^53^ Moreover, the validation study we conducted showed that the search strategy identified almost all eligible RCTs from a bibliographic database search. Searching for RCTs registered in or after 2000 may appear to be a limitation because we did not include most RCTs published before this date without retrospective registration. However, these older RCTs are less relevant for contemporary clinical practice and guideline recommendations given that more comprehensive CVRM care and new therapies have vastly improved patients’ outcomes.^54^

## Conclusions

This systematic review found that representation of patients with CKD in cardiovascular RCTs has not increased in the past 20 years. Cardiovascular RCTs systematically excluded more patients with CKD than expected on safety grounds. Lack of cardiovascular RCTs that reported results for patients with CKD has played a role in the significant evidence gaps in the effectiveness of most CVRM medications for patients with CKD, particularly CKD stages 4 to 5.

## Notes

Supplement 1. **eFigure 1.** Flowchart of Literature Search **eFigure 2.** The Exclusion of Patients With CKD for Different Types of Cardiovascular Medications **eFigure 3.** The Exclusion of Patients With CKD Stratified by Prescribing Recommendations for Exclusion of Patients With CKD **eFigure 4.** The Number of Analyses for Patients With CKD for Different Cardiovascular Outcomes **eFigure 5.** Overview of Individual Studies With Analysis on eGFR or Kidney Replacement Therapy for Major Adverse Cardiovascular Events in Patients With CKD **eFigure 6.** Heat Map of (Subgroup) Analyses for All-Cause Mortality for People With Different Stages of CKD **eFigure 7.** Heat Map of (Subgroup) Analyses for Cardiovascular Mortality for People With Different Stages of CKD **eFigure 8.** Heat Map of (Subgroup) Analyses for Coronary Artery Disease for People With Different Stages of CKD **eFigure 9.** Heat Map of (Subgroup) Analyses for Heart Failure for People With Different Stages of CKD **eFigure 10.** Heat Map of (Subgroup) Analyses for Cerebrovascular Disease for People With Different Stages of CKD **eFigure 11.** Heat Map of (Subgroup) Analyses for Peripheral Arterial Disease for People With Different Stages of CKD **eFigure 12.** Heat Map of (Subgroup) Analyses Kidney Failure for People With Different Stages of CKD

Supplement 2.
Legend.
**eTable 1.** Antihypertensives (Mono) **eTable 2.** Antihypertensives (Multiple) **eTable 3.** Antihypertensives (Other) **eTable 4.** Lipid-Lowering (Mono) **eTable 5.** Lipid-Lowering (Multiple) **eTable 6.** Lipid-Lowering (Other) **eTable 7.** Antiplatelet (Mono) **eTable 8.** Antiplatelet (Multiple) **eTable 9.** Glucose-Lowering (Mono) **eTable 10.** Glucose-Lowering (Multiple)

Supplement 3.
Data Sharing Statement
