# Supplementary material for: Representation of Patients With Chronic Kidney Disease in Clinical Trials of Cardiovascular Disease Medications: A Systematic Review
Source: JAMA Netw Open. 2024 Mar 7;7(3):e240427. doi: 10.1001/jamanetworkopen.2024.0427 (PMC10921252; doi:10.1001/jamanetworkopen.2024.0427)
Supplement: Supplement 1. — eFigure 1. Flowchart of Literature Search eFigure 2. The Exclusion of Patients With CKD for Different Types of Cardiovascular Medications eFigure 3. The Exclusion of Patients With CKD Stratified by Prescribing Recommendations for Exclusion of Patients With CKD eFigure 4. The Number of Analyses for Patients With CKD for Different Cardiovascular Outcomes eFigure 5. Overview of Individual Studies With Analysis on eGFR or Kidney Replacement Therapy for Major Adverse Cardiovascular Events in Patients With CKD eFigure 6. Heat Map of (Subgroup) Analyses for All-Cause Mortality for People With Different Stages of CKD eFigure 7. Heat Map of (Subgroup) Analyses for Cardiovascular Mortality for People With Different Stages of CKD eFigure 8. Heat Map of (Subgroup) Analyses for Coronary Artery Disease for People With Different Stages of CKD eFigure 9. Heat Map of (Subgroup) Analyses for Heart Failure for People With Different Stages of CKD eFigure 10. Heat Map of (Subgroup) Analyses for Cerebrovascular Disease for People With Different Stages of CKD eFigure 11. Heat Map of (Subgroup) Analyses for Peripheral Arterial Disease for People With Different Stages of CKD eFigure 12. Heat Map of (Subgroup) Analyses Kidney Failure for People With Different Stages of CKD [file jamanetwopen-e240427-s001.pdf]

# Supplemental Online Content

Colombijn JMT, Idema DL, van Beem S, et al. Representation of patients with chronic kidney disease in clinical trials of cardiovascular disease medications: a systematic review. *JAMA Netw Open*. 2024;7(2):e240427. doi:10.1001/jamanetworkopen.2024.0427

**eFigure 1.** Flow Chart of Literature Search
**eFigure 2.** The Exclusion of Patients With CKD for Different Types of Cardiovascular Medications
**eFigure 3.** The Exclusion of Patients With CKD Stratified by Prescribing Recommendations for Exclusion of Patients With CKD
**eFigure 4.** The Number of Analyses for Patients With CKD for Different Cardiovascular Outcomes
**eFigure 5.** Overview of Individual Studies With Analysis on eGFR or Kidney Replacement Therapy for Major Adverse Cardiovascular Events in Patients With CKD
**eFigure 6.** Heat Map of (Subgroup) Analyses for All-Cause Mortality for People With Different Stages of CKD
**eFigure 7.** Heat Map of (Subgroup) Analyses for Cardiovascular Mortality for People With Different Stages of CKD
**eFigure 8.** Heat Map of (Subgroup) Analyses for Coronary Artery Disease for People With Different Stages of CKD
**eFigure 9.** Heat Map of (Subgroup) Analyses for Heart Failure for People With Different Stages of CKD
**eFigure 10.** Heat Map of (Subgroup) Analyses for Cerebrovascular Disease for People With Different Stages of CKD
**eFigure 11.** Heat Map of (Subgroup) Analyses for Peripheral Arterial Disease for People With Different Stages of CKD
**eFigure 12.** Heat Map of (Subgroup) Analyses Kidney Failure for People With Different Stages of CKD

This supplemental material has been provided by the authors to give readers additional information about their work.

**eFigure 1:** Flow chart of literature search.

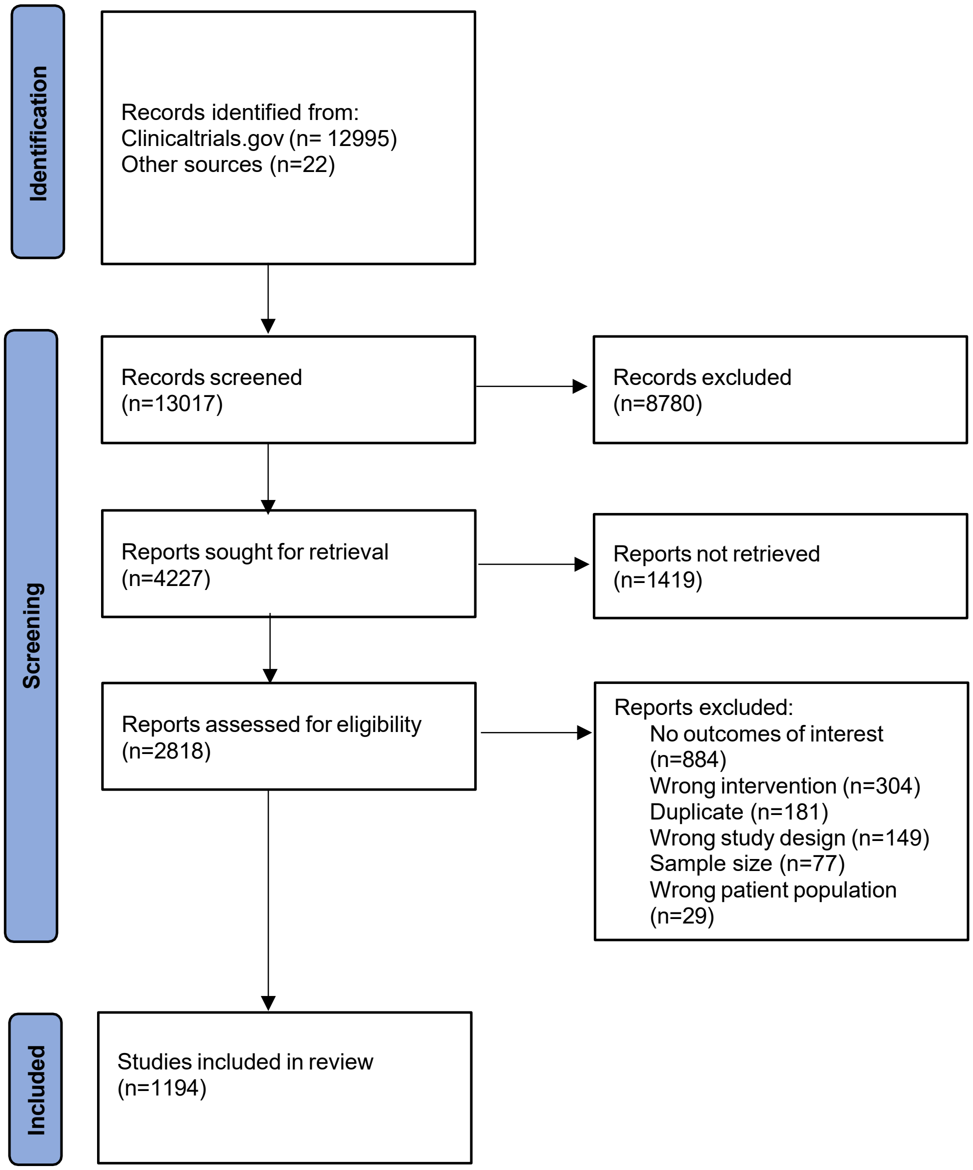


© 2024 Colombijn JMT et al. *JAMA Network Open*

**eFigure 2:** The exclusion of patients with CKD for different types of cardiovascular medications

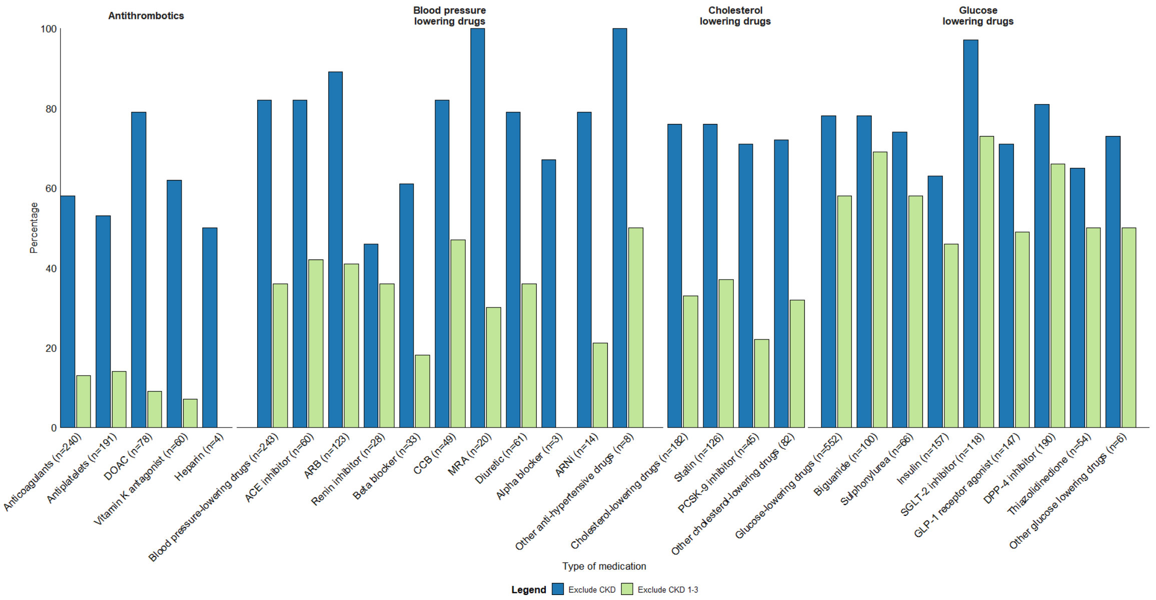


Abbreviations: ACE inhibitor, angiotensin-converting enzyme inhibitor; ARB, angiotensin receptor blocker; CCB, calcium channel blocker; CKD, chronic kidney disease; DOAC, direct oral anticoagulants; DPP-4 receptor agonist, dipeptidyl-peptidase-4 inhibitors; eGFR, estimated glomerular filtration rate; GLP-1 receptor agonist, glucagon-like peptide-1 receptor agonist; MRA, mineralocorticoid receptor antagonist; PSCK-9 inhibitor, proprotein convertase subtilisin/kexin type 9 inhibitor; SCr, serum creatinine; SGLT-2 inhibitor, sodium-glucose cotransporter-2 inhibitor.

© 2024 Colombijn JMT et al. *JAMA Network Open*

**eFigure 3:** The exclusion of patients with CKD stratified by prescribing recommendations for patients with CKD

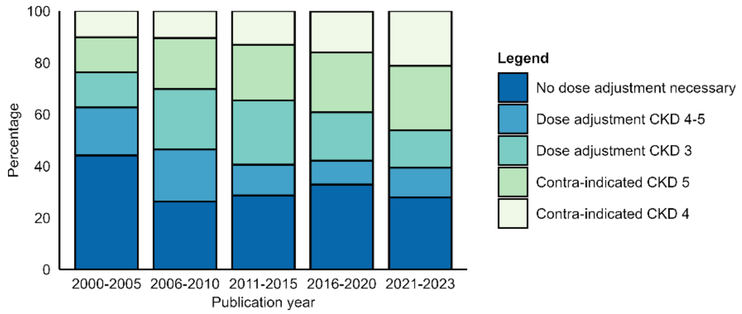


Abbreviations: CKD, chronic kidney disease

**eFigure 4:** the number of analyses for patients with CKD for different cardiovascular outcomes

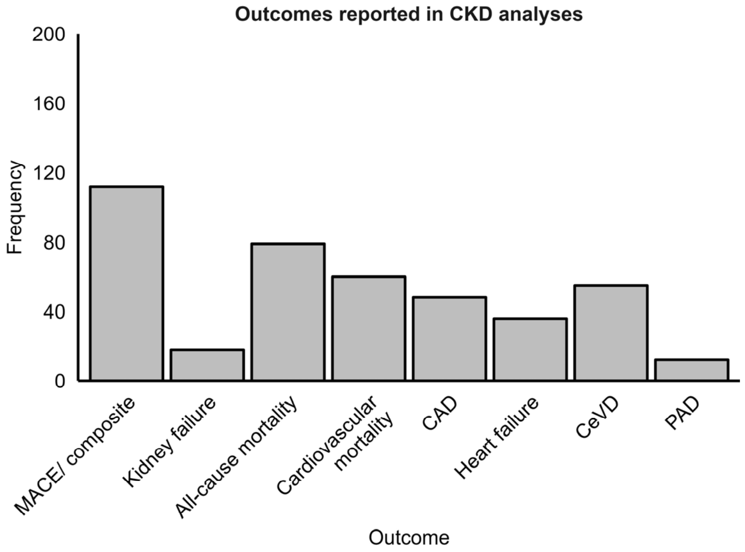


Abbreviatons: CAD, coronary artery disease; CeVD, cerebrovascular disease; CKD, chronic kidney disease; MACE, major adverse cardiovascular event; PAD, peripheral arterial disease.

© 2024 Colombijn JMT et al. *JAMA Network Open*

**eFigure 5:** Overview of individual studies with analysis on eGFR or kidney replacement therapy for major adverse cardiovascular events in patients with CKD. Panel A illustrates analyses for antithrombotics; panel B for blood-pressure-lowering drugs; panel C for cholesterol-lowering drugs; panel D for glucose-lowering drugs; and panel E for polypills.

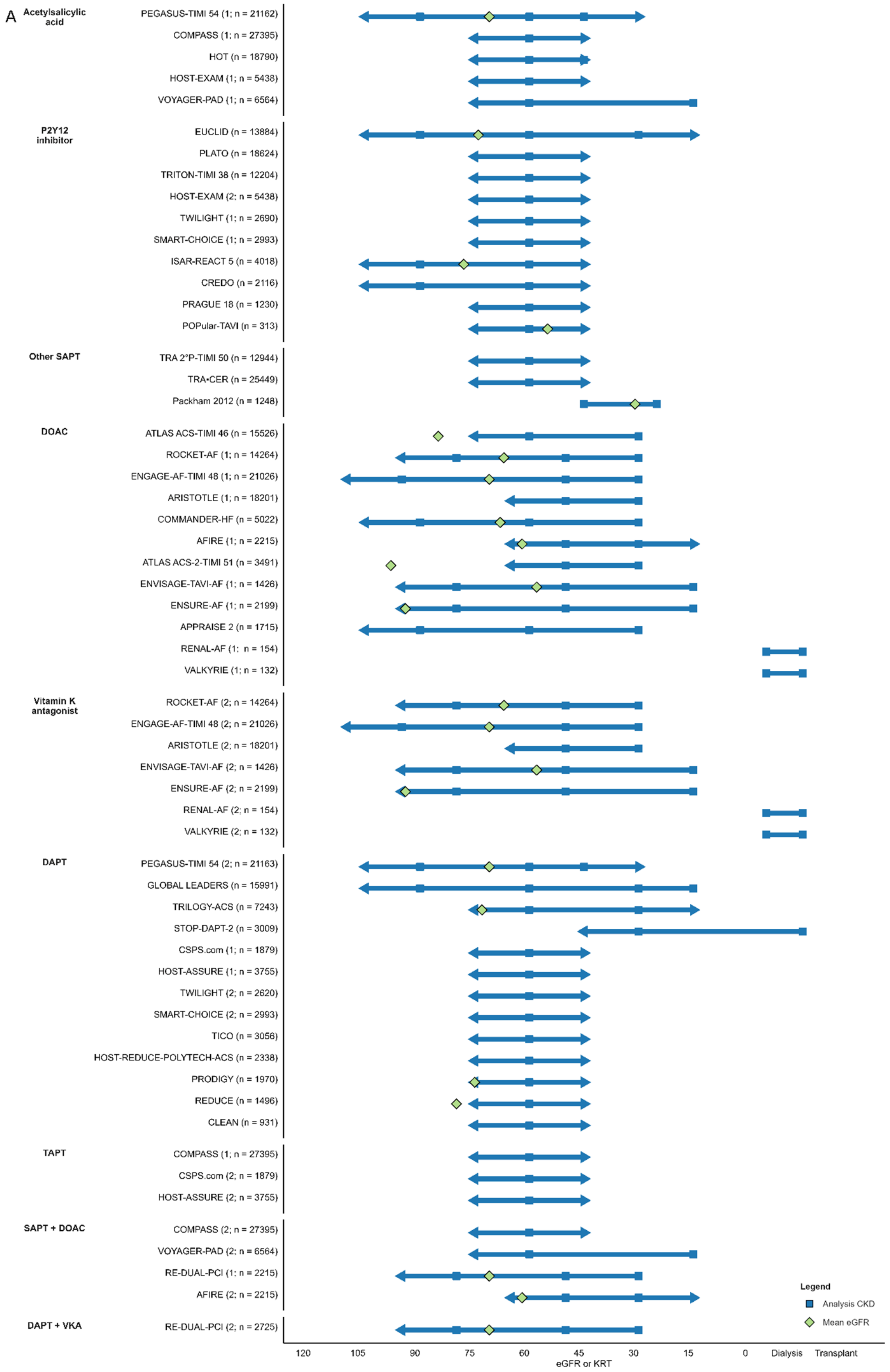


© 2024 Colombijn JMT et al. *JAMA Network Open*

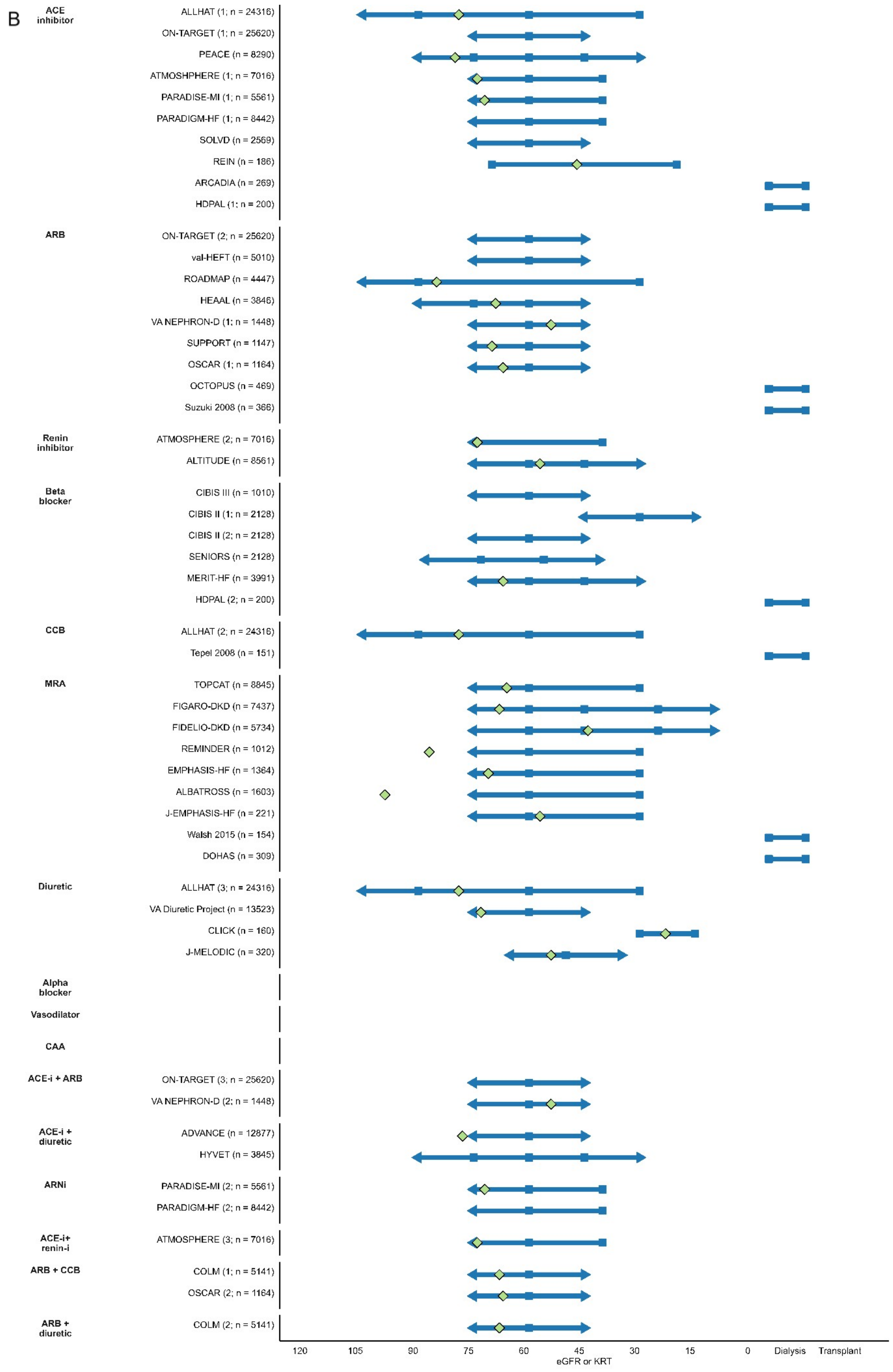


© 2024 Colombijn JMT et al. *JAMA Network Open*

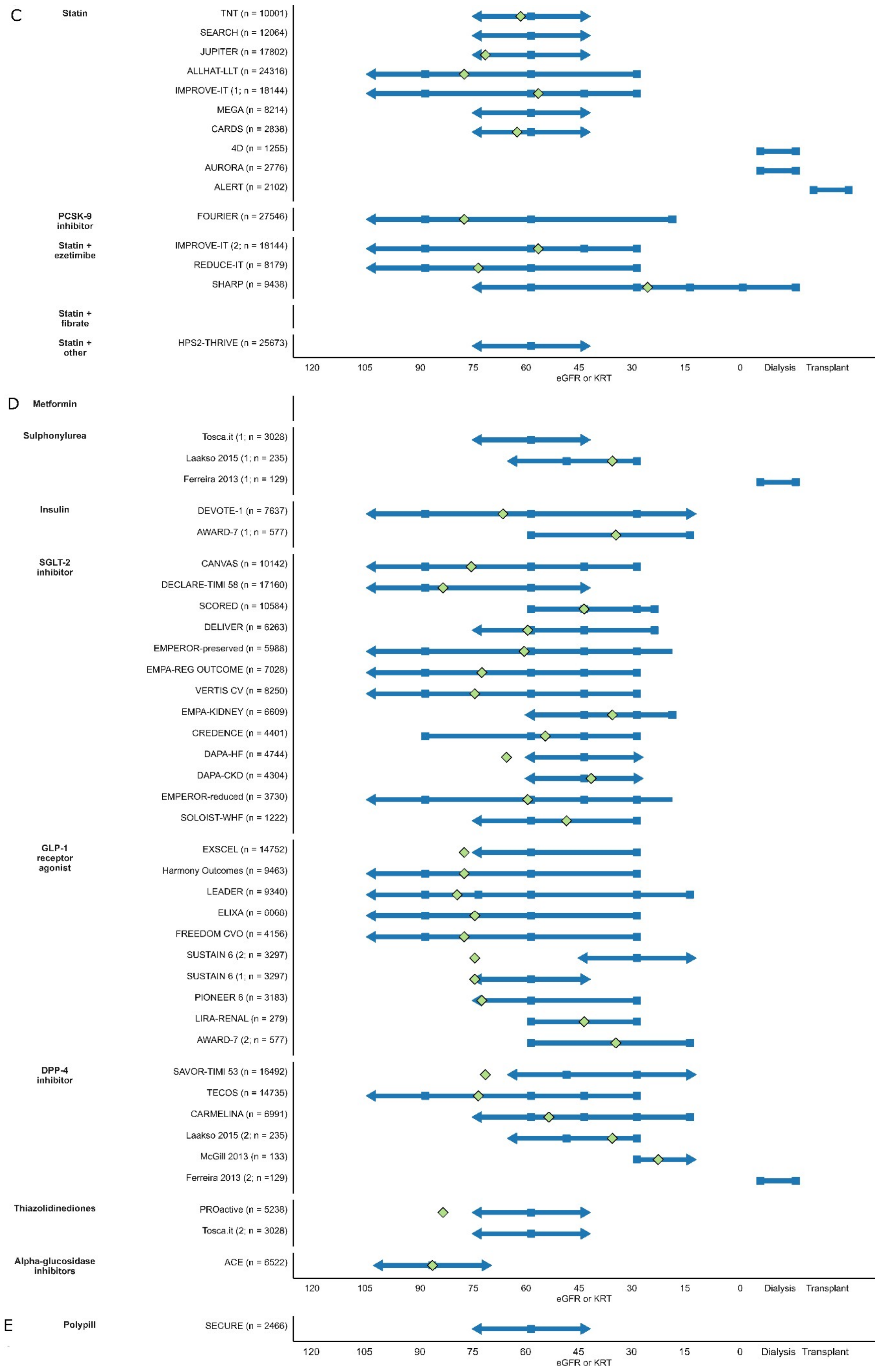


© 2024 Colombijn JMT et al. *JAMA Network Open*

Abbreviations:

ACE-inhibitor/ACE-I, angiotensin-converting enzyme; ARB, angiotensin receptor blocker; CAA, centrally-acting antihypertensive; CCB, calcium channel blocker; CKD, chronic kidney disease; DAPT, double antiplatelet therapy; DOAC, direct oral anticoagulants; DPP-4 inhibitor, dipeptidyl-peptidase-4 inhibitor; eGFR, estimated glomerular filtration rate; GLP-1 receptor agonist, glucagon-like peptide-1 receptor agonist; KRT, kidney replacement therapy; MRA, mineralocorticoid receptor antagonist; PCSK-9 inhibitor, proprotein convertase subtilisin/kexin type 9 inhibitor; SAPT, single antiplatelet therapy; SGLT-2 inhibitor, sodium-glucose cotransporter-2 inhibitor; TAPT, triple antiplatelet therapy; VKA, vitamin K antagonist.

Each line represents a comparison arm in an RCT. Each segment on the line (regardless of type of arrowhead) represents one subgroup analysis. Analyses with strata for which the upper and lower boundaries are specified have a flat arrowhead while RCTs which split strata on an eGFR higher or lower than a certain threshold have pointed arrowheads. These arrows have conveniently been given a length of 15 ml/min/1.73m2 but this length does not represent the upper or lower boundaries of the strata. The diamonds represent the mean eGFR reported in studies. This eGFR can lie outside the strata for which analyses have been performed (e.g. the REMINDER trial) or absent if no mean eGFR was reported (e.g. the ONTARGET trial). RCTs are represented once if the compare drugs within the same drug class (e.g. ace-inhibitors) or placebo and more than once if they compare drugs of different drug classed (e.g. ace-inhibitors vs angiotensin receptor blockers). RCTs which are more than once on the figure have the number in parentheses behind the name of the RCT. For example, the PEGUSUS- TIMI 54 trial, which compared acetylsalicylic acid + rivaroxaban with acetylsalicylic acid, performed a stratified analysis for participants with an eGFR >90, 60-90, 45-60, and <45 ml/min/1.73m2 and the participants in this RCTs had a mean eGFR of 71 ml/min/1.73m2.

© 2024 Colombijn JMT et al. *JAMA Network Open*

**eFigure 6:** Heat map of (subgroup) analyses for all-cause mortality for people with different stages of CKD.

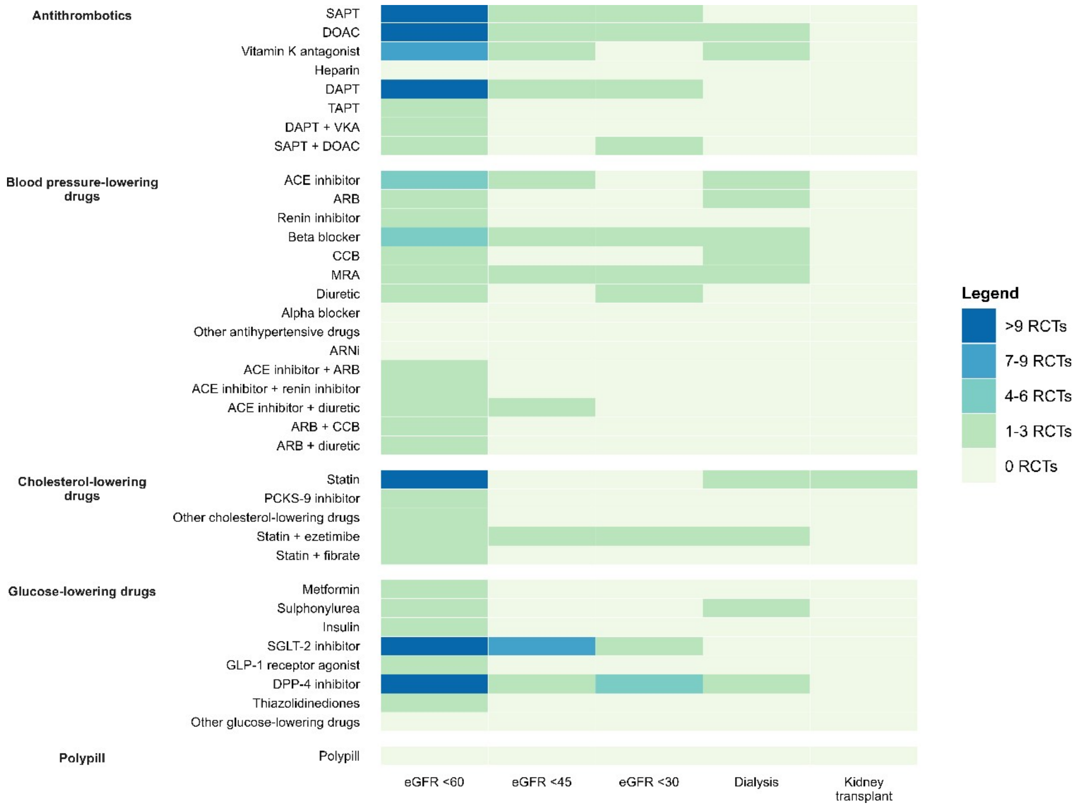


Abbreviations: ACE-inhibitor, angiotensin-converting enzyme inhibitor; ARB, angiotensin receptor blocker; ARNi, angiotensin receptor-neprilysin inhibitor; CCB, calcium channel blocker; DAPT, double antiplatelet therapy; DOAC, direct oral anticoagulant; DPP-4 inhibitor, dipeptidyl-peptidase-4 inhibitors; eGFR, estimated glomerular filtration rate; GLP-1 receptor agonist, glucagon-like peptide-1 receptor agonist; MRA, mineralocorticoid receptor antagonist; PCSK-9 inhibitor, Proprotein convertase subtilisin/kexin type 9 inhibitor; SAPT, single antiplatelet therapy; SGLT-2 inhibitor, sodium-glucose cotransporter-2 inhibitor; TAPT, triple antiplatelet therapy; VKA, vitamin K antagonist.

© 2024 Colombijn JMT et al. *JAMA Network Open*

**eFigure 7:** Heat map of (subgroup) analyses for cardiovascular mortality for people with different stages of CKD.

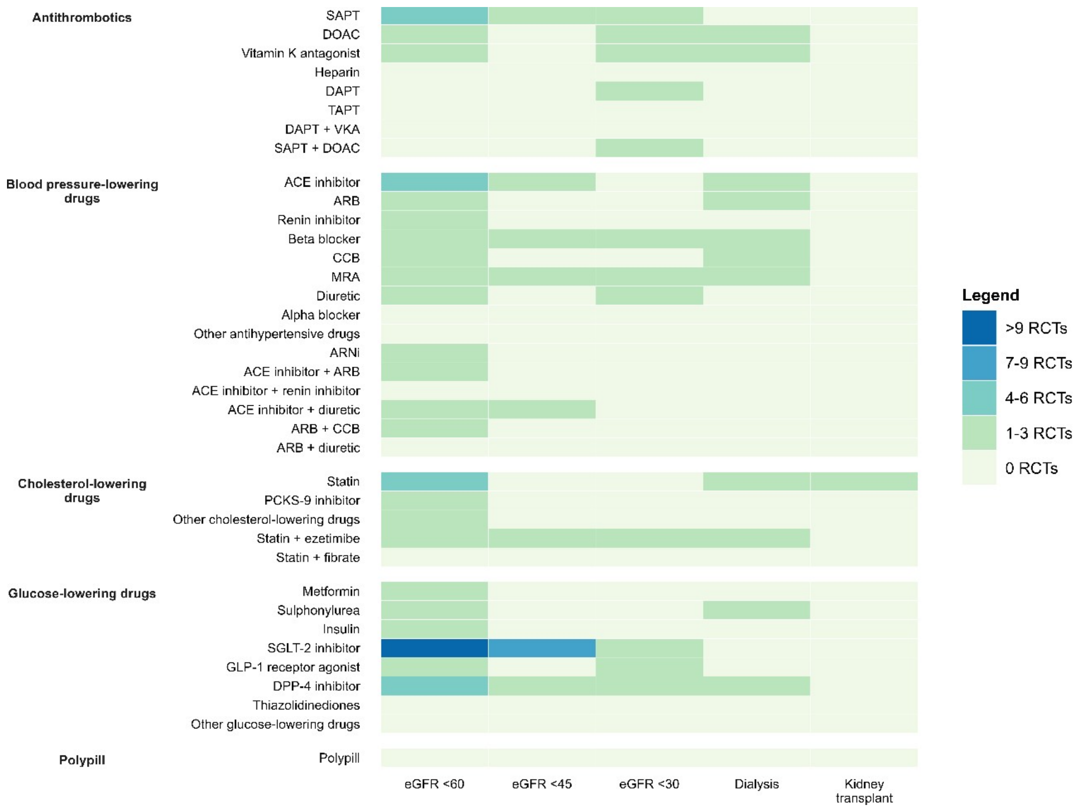


Abbreviations: ACE-inhibitor, angiotensin-converting enzyme inhibitor; ARB, angiotensin receptor blocker; ARNi, angiotensin receptor-neprilysin inhibitor; CCB, calcium channel blocker; DAPT, double antiplatelet therapy; DOAC, direct oral anticoagulant; DPP-4 inhibitor, dipeptidyl-peptidase-4 inhibitors; eGFR, estimated glomerular filtration rate; GLP-1 receptor agonist, glucagon-like peptide-1 receptor agonist; MRA, mineralocorticoid receptor antagonist; PCSK-9 inhibitor, Proprotein convertase subtilisin/kexin type 9 inhibitor; SAPT, single antiplatelet therapy; SGLT-2 inhibitor, sodium-glucose cotransporter-2 inhibitor; TAPT, triple antiplatelet therapy; VKA, vitamin K antagonist.

© 2024 Colombijn JMT et al. *JAMA Network Open*

**eFigure 8:** Heat map of (subgroup) analyses for coronary artery disease for people with different stages of CKD.

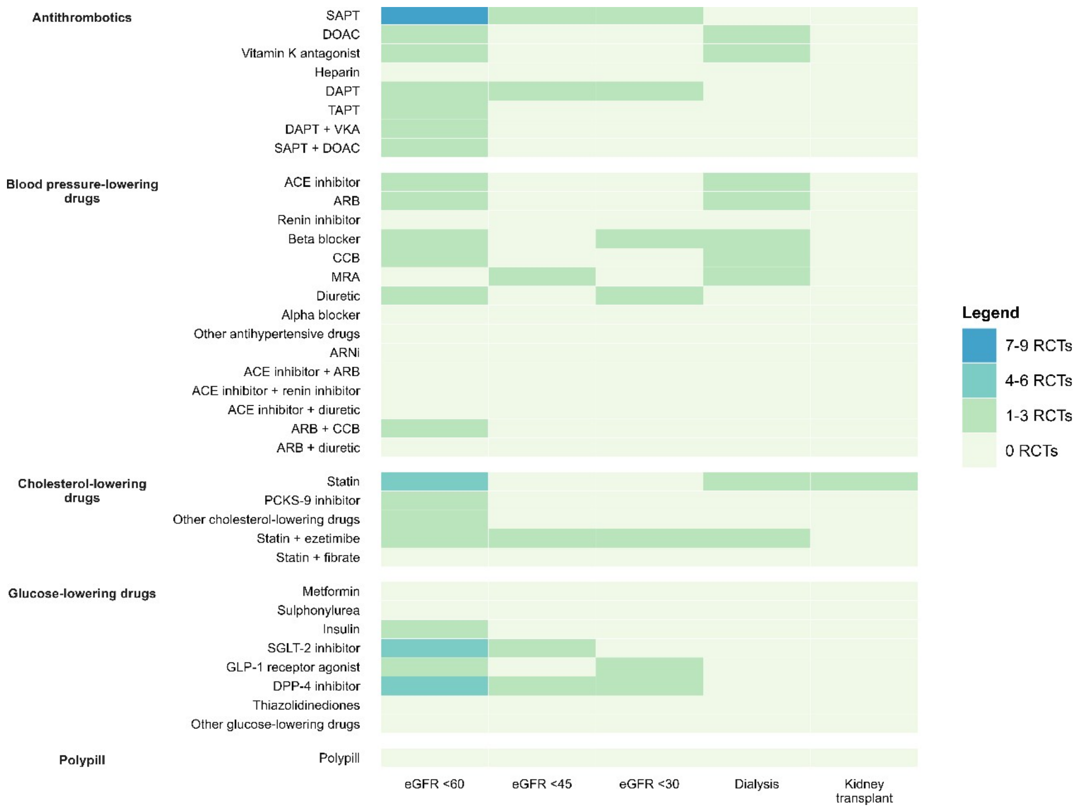


Abbreviations: ACE-inhibitor, angiotensin-converting enzyme inhibitor; ARB, angiotensin receptor blocker; ARNi, angiotensin receptor-neprilysin inhibitor; CCB, calcium channel blocker; DAPT, double antiplatelet therapy; DOAC, direct oral anticoagulant; DPP-4 inhibitor, dipeptidyl-peptidase-4 inhibitors; eGFR, estimated glomerular filtration rate; GLP-1 receptor agonist, glucagon-like peptide-1 receptor agonist; MRA, mineralocorticoid receptor antagonist; PCSK-9 inhibitor, Proprotein convertase subtilisin/kexin type 9 inhibitor; SAPT, single antiplatelet therapy; SGLT-2 inhibitor, sodium-glucose cotransporter-2 inhibitor; TAPT, triple antiplatelet therapy; VKA, vitamin K antagonist.

© 2024 Colombijn JMT et al. *JAMA Network Open*

**eFigure 9:** Heat map of (subgroup) analyses for heart failure for people with different stages of CKD.

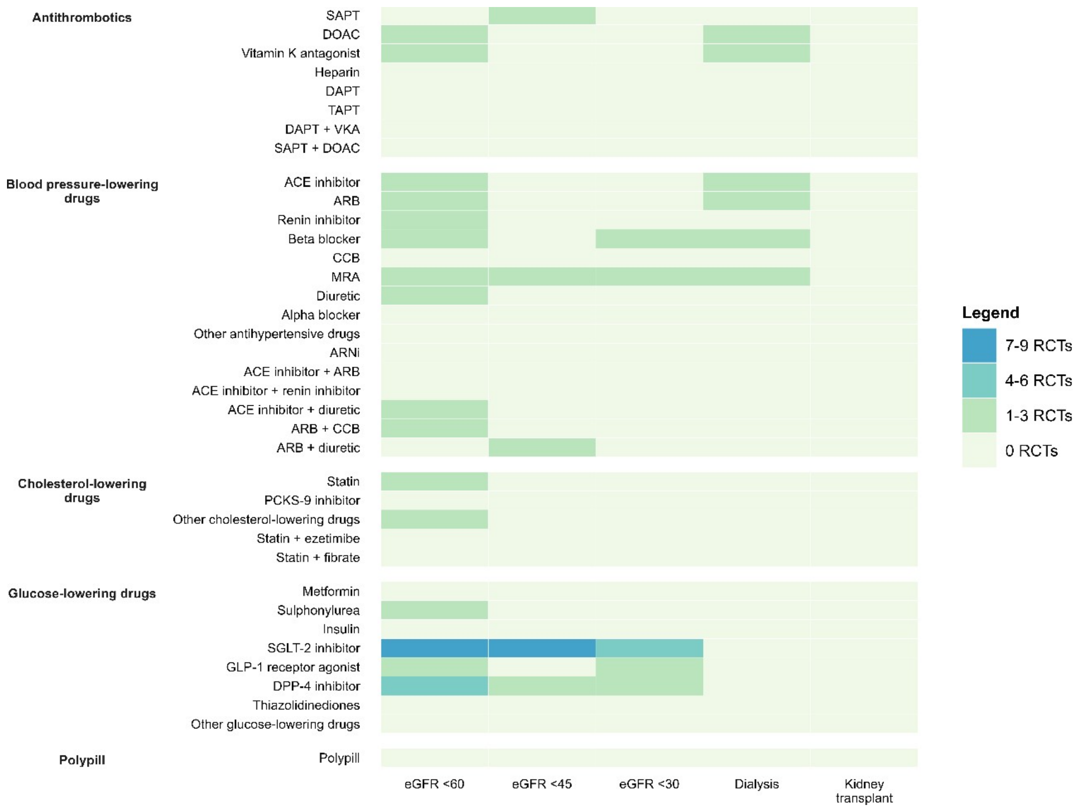


Abbreviations: ACE-inhibitor, angiotensin-converting enzyme inhibitor; ARB, angiotensin receptor blocker; ARNi, angiotensin receptor-neprilysin inhibitor; CCB, calcium channel blocker; DAPT, double antiplatelet therapy; DOAC, direct oral anticoagulant; DPP-4 inhibitor, dipeptidyl-peptidase-4 inhibitors; eGFR, estimated glomerular filtration rate; GLP-1 receptor agonist, glucagon-like peptide-1 receptor agonist; MRA, mineralocorticoid receptor antagonist; PCSK-9 inhibitor, Proprotein convertase subtilisin/kexin type 9 inhibitor; SAPT, single antiplatelet therapy; SGLT-2 inhibitor, sodium-glucose cotransporter-2 inhibitor; TAPT, triple antiplatelet therapy; VKA, vitamin K antagonist.

© 2024 Colombijn JMT et al. *JAMA Network Open*

**eFigure 10:** Heat map of (subgroup) analyses for cerebrovascular disease for people with different stages of CKD.

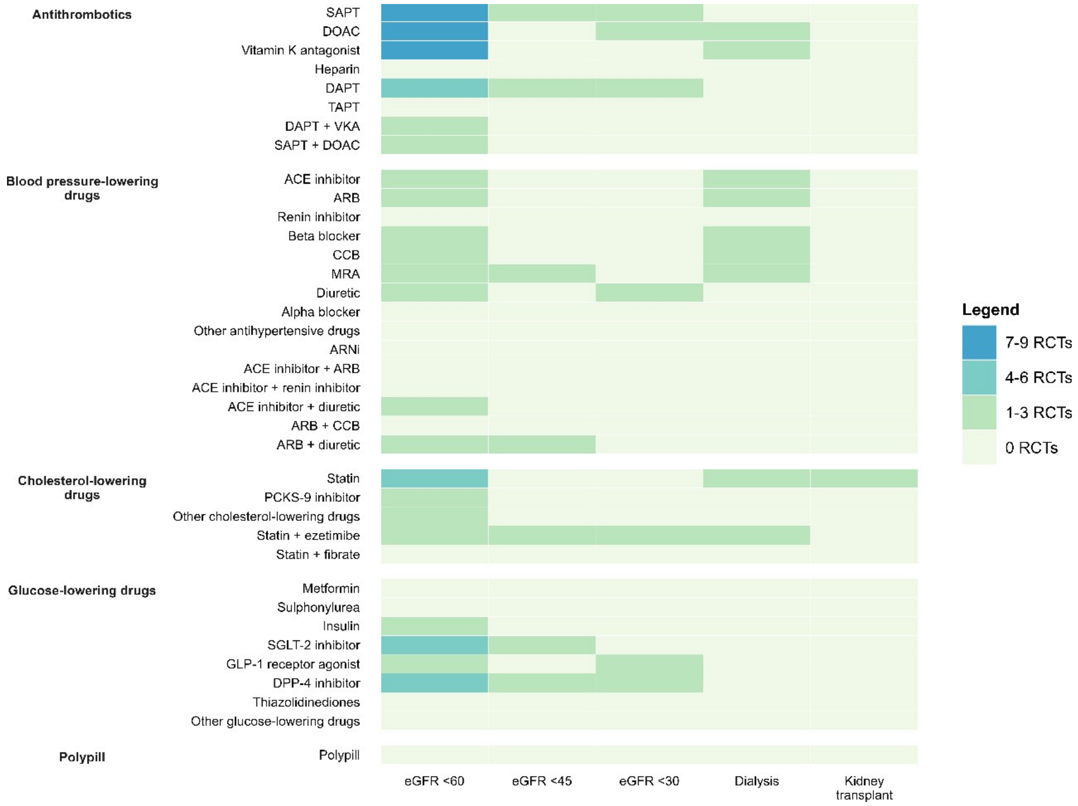


Abbreviations: ACE-inhibitor, angiotensin-converting enzyme inhibitor; ARB, angiotensin receptor blocker; ARNi, angiotensin receptor-neprilysin inhibitor; CCB, calcium channel blocker; DAPT, double antiplatelet therapy; DOAC, direct oral anticoagulant; DPP-4 inhibitor, dipeptidyl-peptidase-4 inhibitors; eGFR, estimated glomerular filtration rate; GLP-1 receptor agonist, glucagon-like peptide-1 receptor agonist; MRA, mineralocorticoid receptor antagonist; PCSK-9 inhibitor, Proprotein convertase subtilisin/kexin type 9 inhibitor; SAPT, single antiplatelet therapy; SGLT-2 inhibitor, sodium-glucose cotransporter-2 inhibitor; TAPT, triple antiplatelet therapy; VKA, vitamin K antagonist.

© 2024 Colombijn JMT et al. *JAMA Network Open*

**eFigure 11:** Heat map of (subgroup) analyses for peripheral arterial disease for people with different stages of CKD.

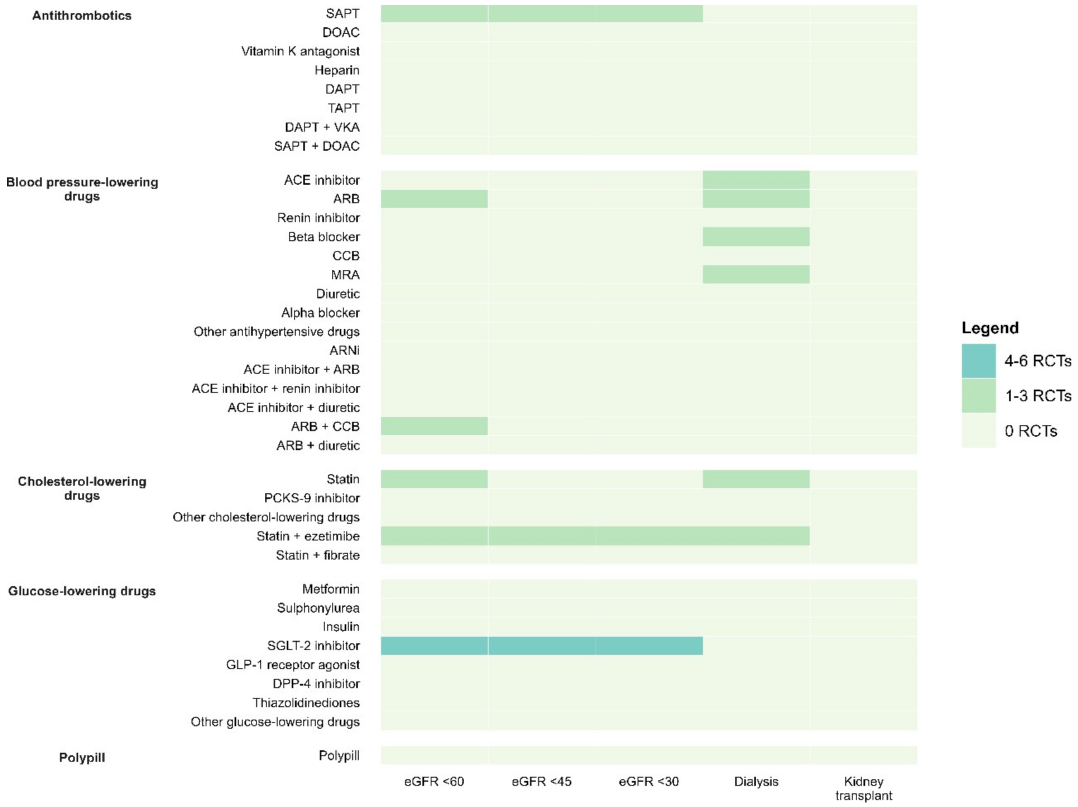


Abbreviations: ACE-inhibitor, angiotensin-converting enzyme inhibitor; ARB, angiotensin receptor blocker; ARNi, angiotensin receptor-neprilysin inhibitor; CCB, calcium channel blocker; DAPT, double antiplatelet therapy; DOAC, direct oral anticoagulant; DPP-4 inhibitor, dipeptidyl-peptidase-4 inhibitors; eGFR, estimated glomerular filtration rate; GLP-1 receptor agonist, glucagon-like peptide-1 receptor agonist; MRA, mineralocorticoid receptor antagonist; PCSK-9 inhibitor, Proprotein convertase subtilisin/kexin type 9 inhibitor; SAPT, single antiplatelet therapy; SGLT-2 inhibitor, sodium-glucose cotransporter-2 inhibitor; TAPT, triple antiplatelet therapy; VKA, vitamin K antagonist.

© 2024 Colombijn JMT et al. *JAMA Network Open*

**eFigure 12:** Heat map of (subgroup) analyses kidney failure for people with different stages of CKD.

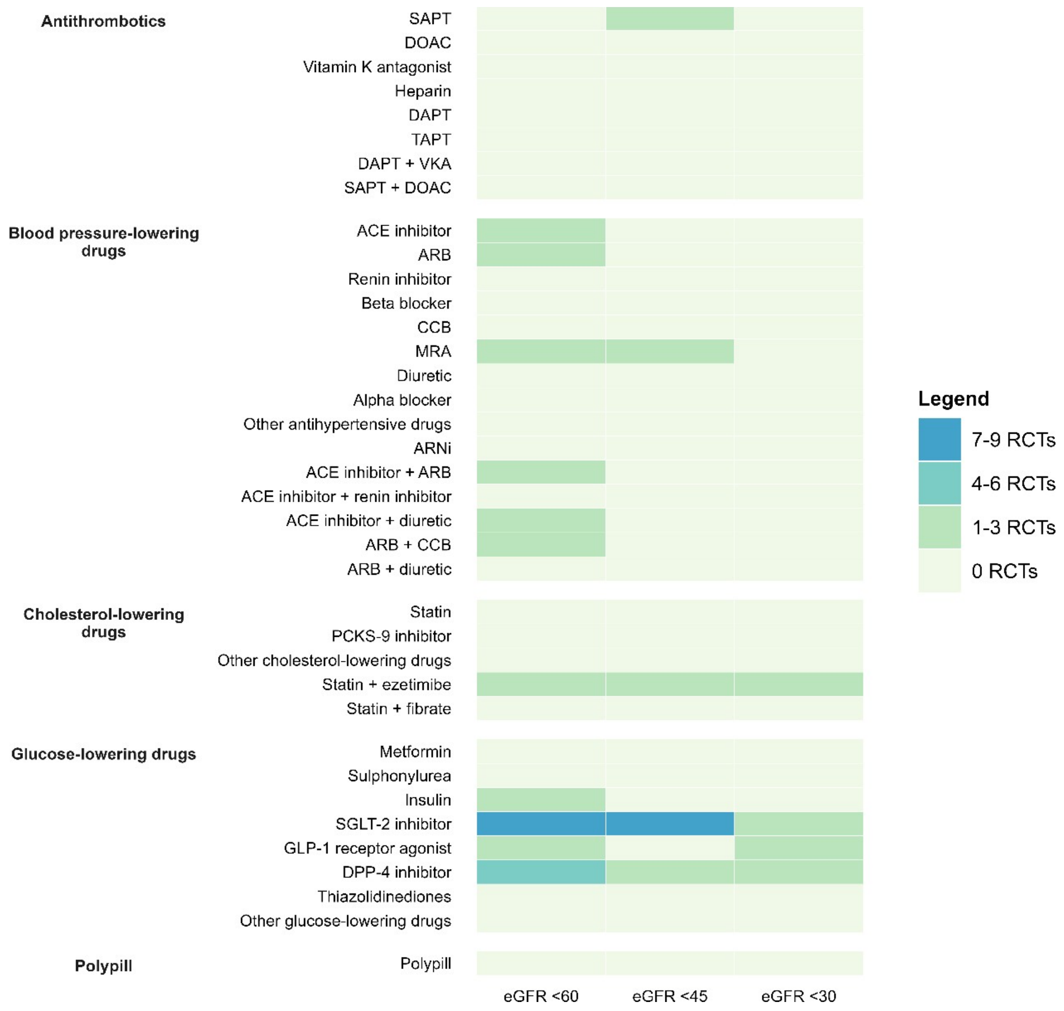


Abbreviations: ACE-inhibitor, angiotensin-converting enzyme inhibitor; ARB, angiotensin receptor blocker; ARNi, angiotensin receptor-neprilysin inhibitor; CCB, calcium channel blocker; DAPT, double antiplatelet therapy; DOAC, direct oral anticoagulant; DPP-4 inhibitor, dipeptidyl-peptidase-4 inhibitors; eGFR, estimated glomerular filtration rate; GLP-1 receptor agonist, glucagon-like peptide-1 receptor agonist; MRA, mineralocorticoid receptor antagonist; PCSK-9 inhibitor, Proprotein convertase subtilisin/kexin type 9 inhibitor; SAPT, single antiplatelet therapy; SGLT-2 inhibitor, sodium-glucose cotransporter-2 inhibitor; TAPT, triple antiplatelet therapy; VKA, vitamin K antagonist.

© 2024 Colombijn JMT et al. *JAMA Network Open*
