# Supplementary material for: Representation of Patients With Chronic Kidney Disease in Clinical Trials of Cardiovascular Disease Medications: A Systematic Review
Source: JAMA Netw Open. 2024 Mar 7;7(3):e240427. doi: 10.1001/jamanetworkopen.2024.0427 (PMC10921252; doi:10.1001/jamanetworkopen.2024.0427)
Supplement: Supplement 3. — Data Sharing Statement [file jamanetwopen-e240427-s003.pdf]

Data Sharing Statement

Colombijn. Representation of Patients With Chronic Kidney Disease in Clinical Trials of Cardiovascular Disease Medications. *JAMA Netw Open*. Published March 07, 2024. doi:10.1001/jamanetworkopen.2024.0427

Data
Data available: Yes
Data types: Data (not involving human participants)
How to access data: All data in this review are based on publicly available articles. Dataset and syntax are available upon request
When available: With publication

Supporting Documents
Document types: Statistical/analytic code
How to access documents: All data in this review are based on publicly available articles. Dataset and syntax are available upon request
When available: With publication

Additional Information
Who can access the data: All data in this review are based on publicly available articles. Dataset and syntax are available upon request
Types of analyses: All data in this review are based on publicly available articles. Dataset and syntax are available upon request
Mechanisms of data availability: All data in this review are based on publicly available articles. Dataset and syntax are available upon request
